# Exploratory Analysis of the Inhibitory Effects of Propranolol on NLRP3 and Pyrin Inflammasomes

**DOI:** 10.1002/acr2.90133

**Published:** 2026-08-23

**Authors:** Renske J. de Jong, Christopher T. Neullens, Aljoscha Swoboda, Melanie Saers, Philipp Berger, Sudheendra Hebbar Subramanyam, Anastasia Wiener, Kim Ohl, Ralf Weiskirchen, Heidi Noels, Tilmann Kallinich, Julia Guthrie, Christoph Kessel, Klaus Tenbrock

**Affiliations:** ^1^ Department of Pediatrics RWTH Aachen University Aachen Germany; ^2^ Department of Pediatric Rheumatology and Immunology WWU Medical Center (UKM) Münster Münster Germany; ^3^ Institute of Molecular Pathobiochemistry, Experimental Gene Therapy and Clinical Chemistry (IFMPEGKC) RWTH Aachen University Aachen Germany; ^4^ Institute for Molecular Cardiovascular Research (IMCAR) RWTH Aachen University Aachen Germany; ^5^ Department of Pediatric Respiratory Medicine, Immunology and Critical Care Medicine Charité – Universitatmedizin Berlin, Berlin, Germany, German Center for Child and Adolescent Health (DZKJ) Berlin site Germany; ^6^ German Rheumatology Research Center a Leibniz‐Institute (DRFZ) Berlin Germany; ^7^ Ludwig Boltzmann Institute for Network Medicine University of Vienna Vienna Austria; ^8^ Department of Pediatric Rheumatology, Inselspital University of Bern Bern Switzerland

## Abstract

**Objective:**

Propranolol, a nonselective beta receptor blocking agents, impacts cAMP levels and is commonly used to treat hypertension and hemangioma in children and adults. Although there are reports indicating its anti‐inflammatory properties, the exact mechanism is not fully understood.

**Methods:**

Murine and human monocytes and macrophages were exposed to ATP and lipopolysaccharide (LPS), as well as *Clostridioides difficile* toxin A (TcdA) and LPS in the presence or absence of propranolol. Levels of interleukin‐1β (IL‐1β), tumor necrosis factor α, and IL‐6 were then measured. This same process was repeated with monocytes obtained from six different patients with familial Mediterranean fever (FMF). In a murine atherosclerosis model, a model known to be highly impacted by inflammasome activation, propranolol was administered through drinking water, and the size of aortic plaques was measured.

**Results:**

Propranolol was found to decrease the production and secretion of IL‐1β in both murine and human monocytes and macrophages following activation of the NLRP3 inflammasome, accompanied by reduced caspase‐1 levels in human cells. Propranolol treatment effectively prevented IL‐1β production in monocytes from healthy donors and patients with FMF after stimulation with the NLRP3 inflammasome activators LPS and ATP, as well as the pyrin inflammasome activator TcdA. In mice, propranolol treatment also resulted in reduced aortic plaque formation.

**Conclusion:**

This exploratory study suggests that propranolol inhibits both the NLRP3 and pyrin inflammasomes in murine and human monocytes and macrophages. Given its established safety profile and approval for pediatric use, propranolol warrants further investigation as a potential treatment for refractory FMF, particularly in regions with limited access to IL‐1β blockade.

## INTRODUCTION

Propranolol, a widely used nonselective β‐adrenergic receptor antagonist for the treatment of hypertension in children and adults, has also been repurposed for infantile hemangioma.^1^, ^2^ In addition to its cardiovascular effects, propranolol has demonstrated strong anti‐inflammatory activity in several proinflammatory murine models.^3^, ^4^, ^5^ In the present study, we investigated whether propranolol could be a potential therapeutic option for familial Mediterranean fever (FMF).

FMF is the most common monogenic autoinflammatory disease. It is caused by mutations in the *MEFV* gene, which encodes pyrin, and is characterized by recurrent, self‐limited attacks of fever, serositis, and arthritis beginning in childhood.^6^, ^7^ Pyrin is an intracellular pattern‐recognition protein that regulates innate immune responses by sensing disturbances of the actin cytoskeleton and controlling inflammasome activation, leading to caspase‐1‐dependent production of interleukin‐1β (IL‐1β).^8^, ^9^, ^10^

Under physiologic conditions, activation of the pyrin inflammasome is tightly controlled by the RhoA‐PKN1/2 signaling pathway. Active RhoA stimulates the serine‐threonine kinases PKN1 and PKN2, which phosphorylate pyrin and facilitate its interaction with inhibitory 14‐3‐3 proteins, thereby preventing inflammasome assembly. Conversely, inactivation of RhoA, for example by bacterial toxins, leads to pyrin dephosphorylation, dissociation of 14‐3‐3 proteins, and assembly of the pyrin inflammasome through recruitment of the adaptor protein ASC and caspase‐1, culminating in IL‐1β maturation and secretion.^11^

FMF‐associated mutations, predominantly clustered within the B30.2 domain of pyrin, impair this inhibitory phosphorylation mechanism and render pyrin constitutively prone to inflammasome activation.^11^ Consequently, mutant pyrin drives excessive IL‐1β production. Supporting a gain‐of‐function disease mechanism, pyrin knock‐in mice harboring mutant human B30.2 domains develop spontaneous autoinflammation characterized by constitutive caspase‐1 activation and enhanced IL‐1β secretion, whereas pyrin‐deficient mice fail to exhibit an inflammatory phenotype.^12^ Although pyrin functions as the primary sensor in FMF, accumulating evidence suggests functional interplay with the NLRP3 inflammasome, which may further amplify caspase‐1 activation and IL‐1‐mediated inflammation in affected patients.^13^, ^14^

In context of FMF disease pathology targeting β‐adrenergic signaling with propranolol may therefore be relevant, as β‐adrenergic pathways are closely linked to inflammasome activation. Human primary monocytes express both β_1_‐ and β_2_‐adrenergic receptors (β‐ARs), and adrenergic stimulation can modulate the expression of inflammasome components, including NALP1/NLRP1, NLRP3, and ASC, thereby influencing caspase‐1‐dependent IL‐1β maturation.^15^ Notably, before the era of genetic diagnostic testing, adrenergic activation using metaraminol infusion was employed to provoke disease‐like attacks in patients with FMF, thereby supporting the diagnosis.^16^

In the present study, we set out to investigate whether propranolol could reduce IL‐1β production in monocytes and macrophages, and whether these effects are more pronounced in patients with FMF, in whom disease pathology is driven by dysregulated pyrin inflammasome activation. We demonstrate that propranolol inhibits IL‐1β production through suppression of both NLRP3 and pyrin inflammasomes, suggesting its potential repurposing for the treatment of inflammasome‐driven inflammatory diseases. In addition, we show the therapeutic effect of propranolol in a murine NLRP3‐dependent atherosclerosis model.

## PATIENTS AND METHODS

### Study subjects and ethical statement

Healthy donors or patients with FMF (both n = 6; Table S1) were recruited from the Department of Pediatric Rheumatology and Immunology at University Children's Hospital Muenster, Germany. All patients were homozygous for the M694V mutation (M694V^hom^) and clinically inactive at the time of sampling. All patients were receiving colchicine treatment, and two of the six were additionally treated with canakinumab. All study participants and/or their parents provided written informed consent. The study was approved by the local ethics committee.

All animal experiments were approved by local authorities (Landesamt fur Natur, Umwelt und Verbraucherschutz Nordrhein‐Westfalen, Germany; approval number 84‐02.04.2013.A333) and complied with German and EU animal protection laws. Every effort was made to minimize suffering.

### Cell isolation and culture conditions

#### Murine peritoneal macrophages

Wildtype and VAV^CRE^CREB^fl/fl^ mice were sacrificed, and 5 mL of ice‐cold phosphate‐buffered saline (PBS) was injected into the abdomen. The fluid was withdrawn, centrifuged (300*g* for five minutes at 4°C), and the cell pellet was resuspended in RPMI supplemented with 2% penicillin‐streptomycin and 10% bovine calf serum. Peritoneal exudate cells were cultured, and nonadherent cells were removed. Adherent cells were detached by digestion with trypsin (0.5%), washed and plated again 24 hours before incubation with propranolol and inflammasome activators.

#### Human peripheral blood mononuclear cells and monocytes

The peripheral blood mononuclear cell (PBMC) fraction was obtained by density gradient centrifugation of EDTA‐treated whole blood from healthy adults. Primary human monocytes from healthy donors and patients with FMF were isolated from EDTA‐treated whole blood using the EasySep Direct Human Monocyte Isolation Kit (StemCell Technologies) according to the manufacturers’ instructions. Cells were cultured at 25 × 10^4^ cells/100 μL in 96‐well U‐bottom tissue culture plates in RPMI+10% Fetal Calf Serum (FCS), 1% glutamine, 1% PS, and 1% non essential amino acids (NEAS).

#### Modulators of cellular responses

**Figure 1:**
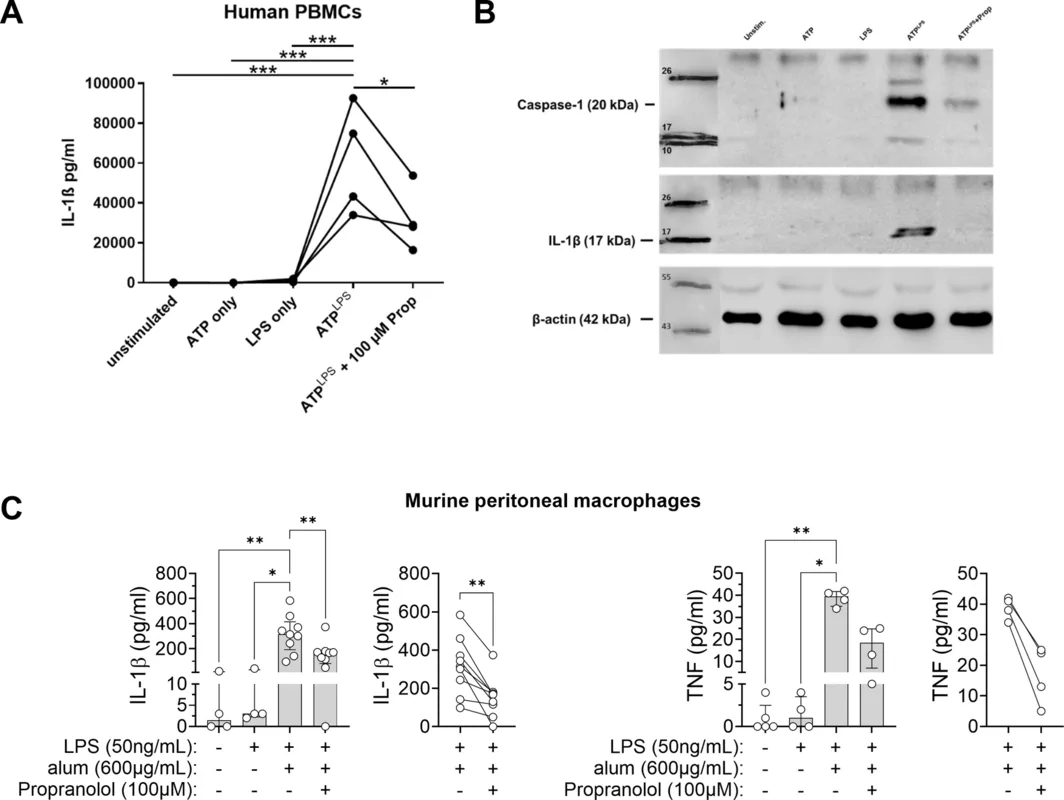
In vitro effects of propranolol on IL‐1β and caspase‐1 expression in human and murine monocytes, PBMCs, and macrophages. (A) Human PBMCs, obtained from four healthy independent donors, were stimulated for four hours with LPS (50 ng/mL) or LPS and ATP (2 mol/mL, added for the last 0.5 hours of stimulation). Where indicated cells were additionally treated with propranolol (100 μM) 0.5 hours before LPS stimulation. Data were analyzed using a pairwise one‐way analysis of variance (RM‐ANOVA) after confirming a normal Gaussian distribution using the Shapiro–Wilk test. (B) PBMCs, stimulated and treated as in (A), were lysed and analyzed by Western blot using antibodies against caspase‐1, IL‐1β, and β‐actin. (C) Peritoneal macrophages from wild type mice were exposed to LPS (18 hours; 50 ng/mL), followed by alum (600 μg/mL, 8 hours), with or without propranolol (100 μM). The release of IL‐1β and TNFα into culture supernatants was quantified by enzyme‐linked immunosorbent assay. Data in bar graphs (median with interquartile range) were analyzed using the Friedman test for multiple paired nonparametric observations, followed by Dunn's multiple comparison test. The connected scatter plots were analyzed using Wilcoxon test. IL, interleukim; LPS, lipopolysaccharide; PBMC, peripheral blood mononuclear cell; TNF, tumor necrosis factor. *P* < 0.05 = *; *P* < 0.01 = **, *P* < 0.001 = ***.

**Figure 2:**
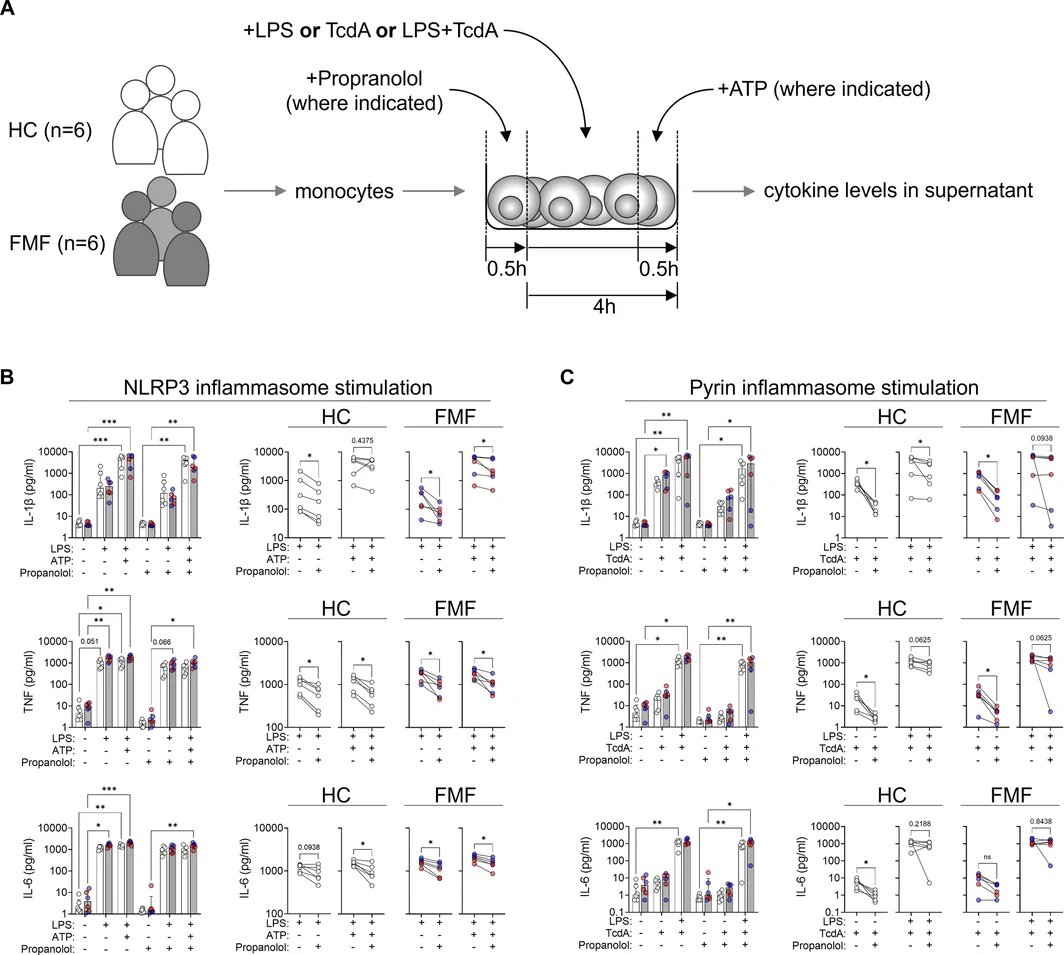
Propranolol reduces cytokine expression associated with both NLRP3 and pyrin inflammasomes in monocytes from HCs and patients with FMF. (A) Experimental setup. (B, C) Monocytes were isolated from freshly drawn blood from HC (n = 6) and treated patients with FMF (n = 6). Cells were stimulated with LPS (50 ng/mL), TcdA (0.5 μg/mL), or combinations of those as indicated for four hours with or without the addition of ATP (0.5 mM) for the last 0.5 hour of stimulation. Where indicated cells were treated with propranolol (100 μM) 0.5 hours before stimulation. Cytokine expression in culture supernatants was quantified by a multiplexed bead array assay. Data in bar graphs (median with interquartile range; white bars: healthy controls; gray bars: patients with FMF) were analyzed by the Friedman test for multiple paired nonparametric observations, followed by Dunn's multiple comparison test. All other data were analyzed by the Wilcoxon test. Data points linked to monocytes isolated from patients with FMF treated with colchicine (n = 3) are colored in blue, data points linked to monocytes isolated from patients with FMF receiving additional treatment with canakinumab (n = 3) are colored in red. FMF, familial Mediterranean fever; HC, healthy control; IL, interleukin; LPS, lipopolysaccharide; TcdA; Clostridium difficile toxin A; TNF, tumor necrosis factor. *P* < 0.05 = *; *P* < 0.01 = **, *P* < 0.001 = ***.

Treatment protocols and concentrations of inflammasome activators are detailed in the figure legends. Briefly, cells were pretreated with propranolol hydrochloride (Dociton, Mibe GmbH) at a final concentration of 1 to 100 μM, 30 minutes before the addition of inflammasome activators. For NLRP3 inflammasome activation, cells were stimulated either with lipopolysaccharide (LPS; 50 ng/mL, 18 hours; *Escherichia coli* O55:B5, Sigma) followed by alum (600 μg/mL, 8 hours; Imject Alum, Thermo Fisher Scientific) as shown in Figure 1, or with LPS (50 ng/mL, 4 hours) followed by ATP (0.5 mM, added after 3.5 hours of LPS stimulation for 30 minutes; ATP disodium salt, Sigma) as shown in Figure 2. Pyrin inflammasomes were activated using Clostridium difficile toxin A (TcdA; 0.5 μg/mL, Enzo) followed by ATP (0.5 mM, added 30 minutes after 3.5 hours of TcdA stimulation). Cell culture supernatants were collected and stored at –20°C for subsequent Luminex analysis.

### Cytokine quantification

To quantify cytokine expression in cell culture supernatants from healthy donors and from the monocytes of patients with FMF, reagents for multiplexed quantification of IL‐1β, IL‐6, and tumor necrosis factor (TNF) were purchased from R&D Systems. The reagents and cell culture supernatants were prepared following the manufacturer's instructions (R&D Systems). Data acquisition and analysis were conducted using a MAGPIX instrument (Merck Millipore) with xPONENT version 4.2 software (Luminex). Additionally, IL‐1β (human IL‐1 beta uncoated enzyme‐linked immunosorbent assay [ELISA] Kit, Invitrogen), TNFα (human TNF alpha uncoated ELISA Kit, Invitrogen), and IL‐6 (human IL‐6 uncoated ELISA Kit, Invitrogen) cytokine concentrations were measured by ELISA following the manufacturer's guidelines.

It should be noted that direct comparison of absolute IL‐1β concentrations across experiments presented in this article is limited, as the studies were performed in separate laboratories (Aachen and Münster) and employed different experimental conditions, including distinct cell‐to‐medium ratios, cytokine quantification methods (ELISA vs Luminex), and cell populations (PBMCs, monocytes, and macrophages).

### Western blot analysis

For Western blot analysis, cells were lysed in Radioimmunoprecipitation Assay (RIPA) buffer containing phosphatase and protease inhibitors. Protein quantification was then conducted. Subsequently, protein lysates were subjected to Sodium Dodecyl Sulfate ‐ Polyacrylamide Gel Electrophoresis (SDS‐PAGE) and transferred to a PVDF membrane. Membranes were blocked with 5% bovine serum albumine (BSA) and incubated with primary antibodies against cleaved caspase‐1 (rabbit p20 polyclonal antibody, Santa Crus Biotechnology), IL‐1β (rabbit monoclonal, 17H18L16, Invitrogen) and β‐actin (mouse monoclonal, AC‐74, Sigma‐Aldrich), followed by secondary antibodies (polycolonal goat anti‐rabbit HRP: P0448 and polyclonal goat anti‐mouse Horseraddish peroxidase (HRP): P0161, both from Dako). A chemiluminescence kit (Millipore) was used to visualize the protein bands.

### Murine model of atherosclerotic plaque development

To model the development of atherosclerosis, apolipoprotein E‐deficient (*Apoe* ^*−/−*^) mice on C57BL/6 background were fed a Western‐type diet for 12 weeks containing 21% fat and 0.2% cholesterol (Sniff TD88137). Mice were maintained under standard specific pathogen–free conditions. Additionally, one group received propranolol via the drinking water (40 mg/kg/day; calculated at 5 mL drinking volume per day). After 12 weeks, the mice were euthanized using ketamine (100 mg/kg) or xylazine (10 mg/kg) anesthesia. Blood was collected by cardiac puncture. A differential blood count was performed using a hematology analyzer. Before isolating the aorta, the circulation was flushed with PBS. Next, the circulation was rinsed with 4% paraformaldehyde containing EDTA and sucrose. Then the aorta was isolated from the ascending aorta to the ileac bifurcation, opened longitudinally, mounted on glass slides, and stained with Oil‐Red‐O *en face* for quantification of lipid deposition.

### In silico mapping

Targets of propranolol, as identified in DrugBank,^17^ include inflammasome‐associated proteins (canonical inflammasome complex [GO:0061702] and cellular responses to IL‐1 [GO:0071347]), and caspases (HUGO Caspase Gene Group).^18^ These targets were mapped onto a protein–protein interactome of 18,068 nodes and 306,914 links. This interactome was compiled from various large‐scale protein interaction data sets including Bioplex, HuRi, Biogrid, Intact, and Hippie.^19^, ^20^, ^21^, ^22^ To evaluate the potential connectivity between propranolol drug targets, the inflammasome, and IL‐1, we analyzed the largest connected component (LCC) of directly connected proteins annotated with propranolol, inflammasome, and IL‐1.

### Statistics

The specific statistical test used for each graph or analysis is detailed in the figure legends. *P* < 0.05 = *; *P* < 0.01 = **, *P* < 0.001 = ***.

## RESULTS

### In vitro inhibition of inflammasome‐dependent cytokine expression upon propranolol treatment

To investigate whether propranolol could reduce IL‐1β production, we stimulated human primary monocytes (Figure S1) and PBMCs (Figure 1A) with LPS and ATP, a combination that activates the NLRP3 inflammasome pathway and induces IL‐1β secretion. Cells were treated with propranolol either before and/or during stimulation. Under these conditions, propranolol induced a dose‐dependent reduction in IL‐1β secretion in human monocytes (Figure S1). A similar suppressive effect on IL‐1β production was observed in human PBMCs following LPS and ATP stimulation (Figure 1A). In addition, propranolol reduced caspase‐1 expression in PBMCs, which is expected to limit IL‐1β maturation and thereby contribute to the decreased IL‐1β output (Figure 1B; Figure S2).

We further examined murine peritoneal macrophages stimulated with LPS and alum, another classic NLRP3 activator, in the absence or presence of propranolol (Figure 1C). Propranolol significantly inhibited IL‐1β and TNFα production in this setting. To test the underlying mechanism, we used CREB‐deficient mice. CREB drives gene expression in response to cAMP.^23^ Notably, propranolol has been reported to increase cAMP levels in hemangioma stem cells.^24^ In our study, CREB‐deficient macrophages exhibited reduced IL‐1β secretion upon stimulation (Figure S3).

### Inhibition of NLRP3 and pyrin inflammasome activity upon propranolol treatment in monocytes from patients with FMF

Dysregulated inflammasome activity and IL‐1 family cytokine expression plays a central role in the pathology of several autoinflammatory diseases. Therefore, we aimed to investigate how propranolol treatment affects inflammatory cytokine expression in monocytes within the context of FMF, a condition characterized by dysregulated pyrin inflammasome activity.^10^

Isolated monocytes from patients with FMF (n = 6) or healthy controls (HCs, n = 6; Table S1) were treated with LPS and ATP as well as LPS and *Clostridioides difficile* toxin A (TcdA) as activators of the NLRP3 and pyrin inflammasomes, respectively (Figure 2A). Stimulation of monocytes from healthy donors and patients with FMF with LPS alone already resulted in a slight up‐regulation of IL‐1β expression, which was reversed by propranolol treatment (Figure 2B, upper panels). When combined with ATP as a second signal for NLRP3 inflammasome activation, there was a significant up‐regulation of IL‐1β expression, which could also be partially blocked by propranolol (Figure 2B, upper panels). In addition to IL‐1β, we also observed a reduction in TNFα and IL‐6 release with propranolol treatment throughout (Figure 2B, central and lower panels).

Activation of the pyrin inflammasome with TcdA induced a significant increase in IL‐1β release from monocytes of patients with FMF compared with HCs (Figure 2C, upper panels). For both groups propranolol could reduce IL‐1β release (Figure 2C, upper panels). Stimulation with both TcdA and LPS further enhanced IL‐1β secretion, largely abrogating the baseline difference between patients with FMF and HCs, yet propranolol still partially reduced IL‐1β release, although this inhibitory effect was more pronounced in cells from healthy donors (Figure 2C, upper panels). Inhibition of TNFα and IL‐6 release by propranolol was more evident in monocytes treated with TcdA alone than in those stimulated with the TcdA/LPS combination (Figure 2B, central and lower panels).

### In silico mapping of propranolol drug targets

**Figure 3:**
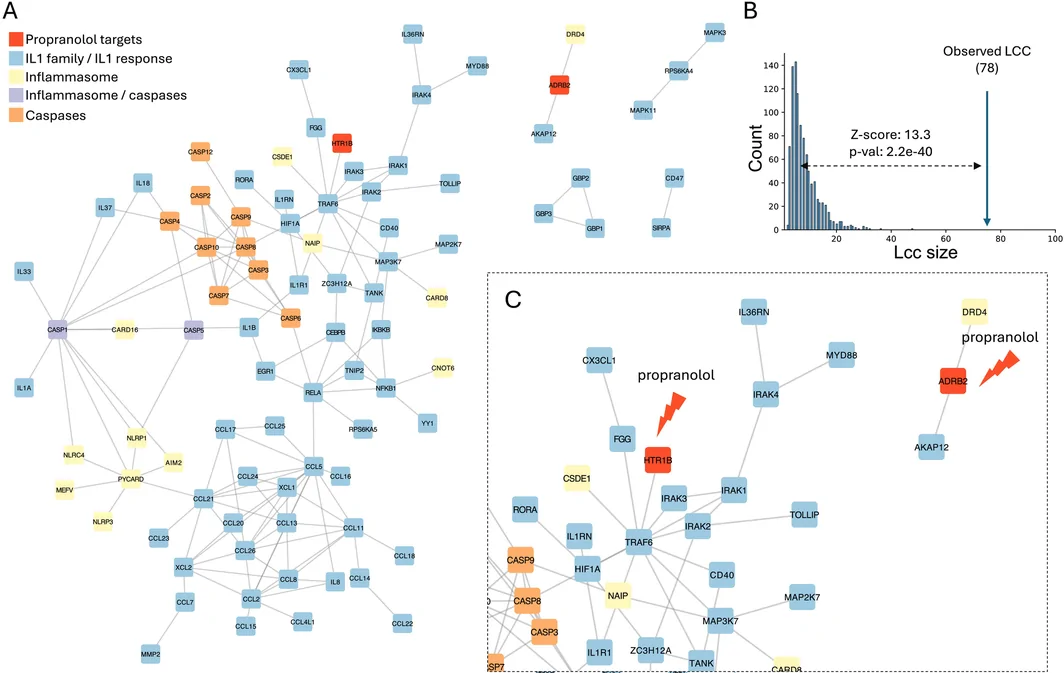
In silico mapping predicts that propranolol can impact inflammasome and IL‐1 signaling related networks. (A) LCCs of the propranolol target (red), inflammasome (yellow and purple), caspase (orange), and IL1 (blue) protein–protein interaction network. Each node represents a protein, and connections between them denote direct physical interactions. (B) The propranolol–inflammasome–caspase–IL1 network is more connected than expected by chance, measured by the size of the LCC. The Z score was determined by comparing the connectivity of the gene sets to 1,000 random permutations of connectivity calculations of gene sets of the same size. (C) Direct connections between propranolol targets (HTR1B and ADRB2), IL‐1, and inflammasome pathways. IL, interleukin; LCC, largest connected component.

Our data indicate that propranolol may have a therapeutically relevant effect through its specific mode of action. To gain a broader understanding of the molecular context of this effect, we conducted network analysis of the molecular interactions of propranolol. In short, we mapped targets of propranolol (identified using DrugBank), inflammasome associated proteins (canonical inflammasome complex [GO:0061702], cellular response to IL‐1 [GO:0071347]), and caspases (HUGO Caspase Gene Group) onto a protein–protein interactome of 18,068 nodes and 306,914 links, compiled from various large‐scale protein interaction data sets (Bioplex, HuRi, Biogrid, Intact, Hippie). To evaluate the potential connectivity of propranolol drug targets, the inflammasome, and IL‐1, we calculated the LCC of directly connected proteins annotated with propranolol, inflammasome, and IL‐1. The LCC included 78 of 143 proteins, forming a highly connected subnetwork compared to random expectation (*z* score: 13.3, empirical *P* value = 2.2 × 10^−40^, Figure 3A and B). Additionally, targets of propranolol were directly linked to inflammasome and IL‐1‐related proteins (Figure 3C). Thus, we were able to demonstrate that the targets of propranolol are located within the same network neighborhood as inflammasome‐related proteins, suggesting propranolol as a potential regulator of inflammasome and IL‐1 pathways using an independent protein interaction data set.

### Propranolol protects from atherosclerotic plaque development

**Figure 4:**
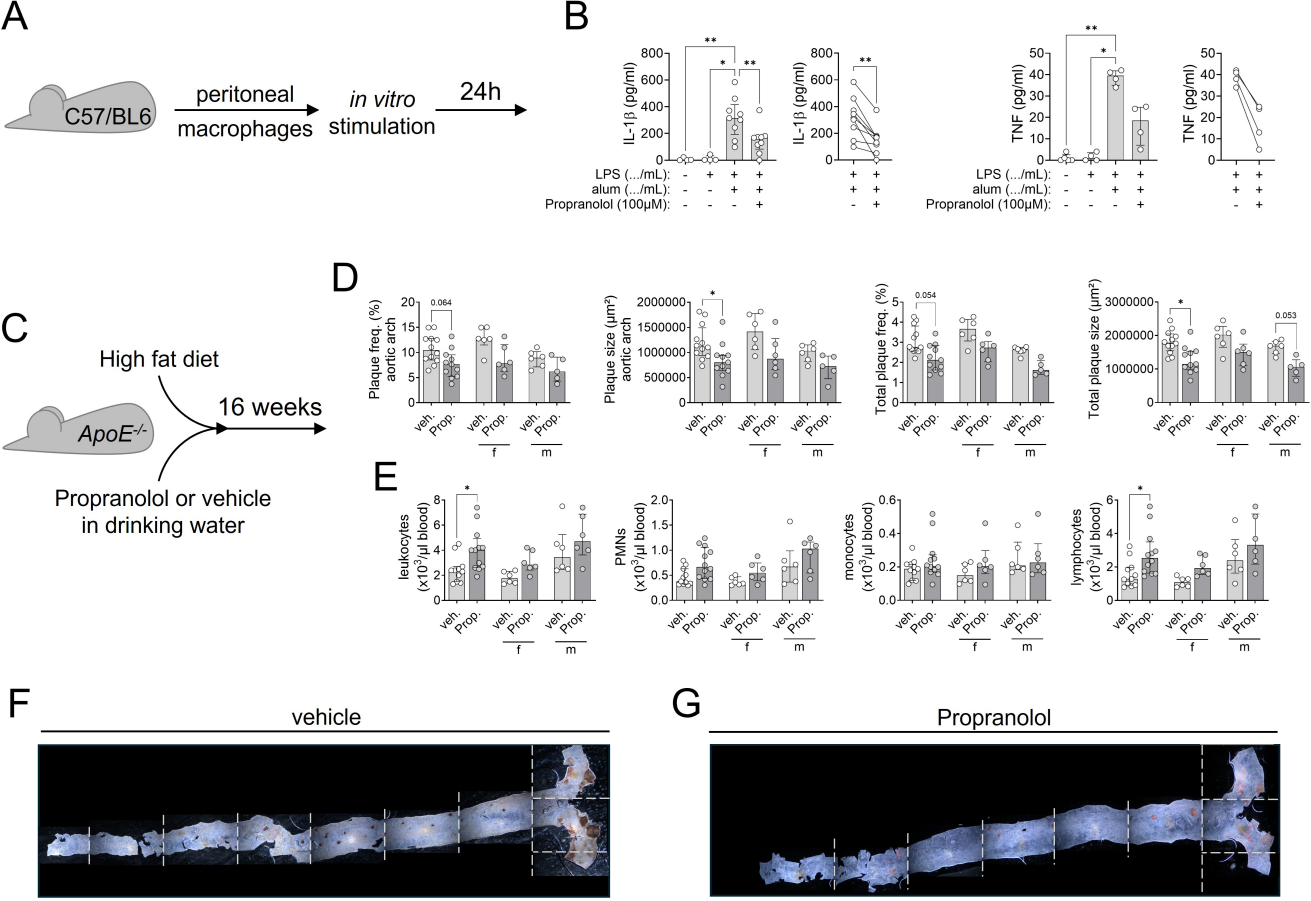
Propranolol protects from atherosclerotic plaque development. (A) *ApoE* ^*−/−*^ mice were fed a high fat diet for 12 weeks, with some receiving additional Prop. treatment (Prop.) via drinking water. Atherosclerotic plaque formation was quantified in (B) the aortic arch and total aorta after aortic en‐face preparation and Oil‐Red‐O lipid staining. (C) Total leukocytes, PMNs, monocytes, and lymphocytes were measured in total blood at the end of the experiment. (D) Representative images of the total aorta were created by combining consecutive pictures as indicated by the dashed lines. The data shown represent means ± SD for m, f, and combined. Two‐way analysis of variance with Sidak's multiple comparisons test was used for analysis. F, females; IL, interleukin; m, males; PMN, polymorphonuclear neutrophil; Prop, propranolol; TNF, tumor necrosis factor. *P* < 0.05 = *; *P* < 0.01 = **, *P* < 0.001 = ***.

We then investigated whether propranolol would also have an effect in vivo and conducted an ApoE or high fat diet (HFD)–induced atherosclerosis mouse model, which is a NLRP3 inflammasome‐dependent model. *ApoE* ^*−/−*^ deficient mice were placed on a HFD (0.2% cholesterol and 21.2% total fat) for 12 weeks (Figure 4A), resulting in the development of atherosclerosis with aortal plaque deposition. The intervention group received propranolol in their drinking water. After 12 weeks, no difference was observed between the body weight of mice with and without treatment of propranolol (Figure S4A). Mice were euthanized and sacrificed, and a histologic analysis of the aorta was performed. Treatment with propranolol led to a decrease in plaque size and frequencies in the aortic arch of mice compared to controls, regardless of gender (Figure 4B and D), whereas plaque size and frequencies in the thoracic, abdominal aorta and aortic root were not affected (Figure 4D; Figure S4C–E). Interestingly, this phenotype persisted despite increased leukocytosis upon propranolol treatment (Figure 4C), which typically correlates with accelerated plaque development.^25^ These findings suggest that propranolol also has an in vivo effect on the NLRP3 inflammasome.

## DISCUSSION

In this study, we demonstrate that propranolol acts as an inhibitor of both the NLRP3 and pyrin inflammasomes, providing a mechanistic basis for the previously reported anti‐inflammatory effects of propranolol.^3^, ^4^, ^5^ Propranolol treatment effectively prevented IL‐1β production in murine peritoneal macrophages and in primary monocytes from healthy human donors as well as patients with FMF. This effect was observed following stimulation with the NLRP3 inflammasome activator combination LPS and ATP, as well as with the pyrin inflammasome activator TcdA and appeared unaffected by underlying treatment of the patients.

Propranolol competes with endogenous catecholamines, such as epinephrine and norepinephrine, for binding to β‐adrenergic receptors, thereby preventing receptor activation. Stimulation of β‐adrenergic receptors can markedly increase intracellular cAMP levels. For example, activation of β_1_‐adrenergic receptors by isoproterenol elevates cAMP and, in the presence of LPS, can synergistically enhance IL‐1β production through a cAMP‐dependent pathway mediated by Protein Kinase A (PKA).^26^ By blocking β‐adrenergic receptors, propranolol interferes with these signaling pathways.

As noted in the Introduction, before the availability of genetic diagnostic testing, adrenergic activation with metaraminol infusion was used to provoke disease‐like attacks in patients with FMF.^16^ Our data now provide a mechanistic explanation for this observation, suggesting that blockade of β_2_‐adrenergic signaling can prevent IL‐1β cleavage.

The role of cAMP in regulating NLRP3 inflammasome activation is both intriguing and controversial. Some studies report that elevated cAMP levels suppress NLRP3 activation.^27^, ^28^

For example, calcium‐sensing receptor signaling promotes NLRP3 inflammasome assembly through intracellular Ca^2+^ flux while simultaneously reducing cellular cAMP levels in mouse bone marrow–derived macrophages (BMDMs) and human PBMCs, suggesting that cAMP acts as an inhibitory signal.^28^ In this context, cAMP can bind directly to NLRP3 to prevent inflammasome assembly, and lowering cAMP levels relieves this inhibition. Conversely, activation of the cAMP–PKA pathway has also been shown to inhibit NLRP3 activity by promoting K63‐linked ubiquitination of NLRP3 in BMDMs.^29^ In our study, we observed decreased NLRP3 inflammasome activation when cAMP signaling was blocked in CREB‐deficient murine peritoneal macrophages. Currently, we cannot fully explain the discrepancy between our findings and previous reports. Notably, propranolol has been shown to increase cAMP levels in hemangioma stem cells.^24^ Future studies are needed to clarify how propranolol‐mediated modulation of cAMP signaling affects NLRP3 inflammasome activity.

Concerning pyrin inflammasome activation, the inhibitory effect of propranolol treatment may be associated with its impact on the actin cytoskeleton. Previous data indicate that activation of β‐AR signaling increases macrophage phagocytosis and migration, two processes that require active cytoskeleton remodeling.^30^ In this setting, pretreatment with propranolol blocked isoproterenol‐enhanced *E coli* internalization, demonstrating a β‐AR‐dependent effect of isoproterenol on phagocytosis. Interestingly, the cAMP activator forskolin enhanced phagocytosis, indicating a role for cAMP in regulating phagocytosis.^30^ In hemangioma, propranolol was shown to block endothelial cell proliferation, migration, and formation of the actin cytoskeleton, coinciding with alterations in vascular endothelial growth factor receptor‐2, p38, and cofilin signaling.^1^ In the context of FMF, actin cytoskeleton reorganization is an important step during pyrin inflammasome activation.^10^ By intervening actin reorganization via cAMP blockade, propranolol could potentially alter pyrin inflammasome activation and prevent subsequent cleavage and release of IL‐1β.

Alongside IL‐1β, we observed that propranolol also prevented the production of TNFα and IL6. The inflammasome does not directly generate TNFα and IL6, however, the pro‐inflammatory cytokine IL‐1β can stimulate the release of other cytokines, explaining the reduction of TNFα, and IL6 alongside IL‐1β.^31^ In addition, propranolol and other beta‐blockers can inhibit NF‐κB and STAT pathways, which drive the transcription of proinflammatory cytokines like IL‐6 and TNFα.^32^

Propranolol is not simply an anti‐inflammatory drug. There have been reports about propranolol‐induced psoriasis.^33^, ^34^, ^35^ In a study by Müller et al using monocytic‐derived Langerhans cells, it was found that NFKB/NF‐κB and MAPK14/p38 activation were necessary for propranolol‐induced IL‐23a secretion, whereas the NLRP3 inflammasome was not essential and cAMP levels did not play a role.^35^ Instead, the authors identified the adaptor protein TRAF6 as a key regulator in the pathophysiology of IL‐23a secretion, as TRAF6 is a signal transducer in the NFKB pathway. Interestingly, TRAF6 was also identified in our network analysis as one of the direct network neighbors of HTR1B, which was identified as one of the propranolol drug targets. TRAF6 is a central adaptor linking TLR/IL‐1 receptor signaling to NF‐κB activation and mitochondrial reactive oxygen species (ROS) generation, which may represent a mechanistic node through which propranolol attenuates inflammasome activity. Given the dependence of the NLRP3 inflammasome on mitochondrial stress signals, ROS production, and metabolic reprogramming, suppression of TRAF6‐mediated pathways could contribute to reduced caspase‐1 activation and IL‐1β maturation.

This study has several limitations. First, we used a murine model of NLRP3‐dependent atherosclerosis rather than an FMF‐specific model to evaluate the therapeutic efficacy of propranolol in vivo. Unfortunately, we were unable to access an appropriate FMF mouse model within the timeframe of this study. Local regulations governing the breeding and use of FMF mouse strains are highly restrictive, and we were therefore unable to establish collaborations that would have enabled testing of propranolol in these models. Nevertheless, we consider the atherosclerosis model a reasonable surrogate, given the well‐established contribution of inflammasome activation to disease progression.^36^, ^37^ This approach allowed us to assess the in vivo effects of propranolol on inflammasome‐driven inflammation.

Second, our clinical cohort comprised only six patients with FMF. Consequently, our findings should be interpreted with caution and regarded as exploratory. Larger, adequately powered studies are required to validate these observations and to further define the therapeutic potential of propranolol in FMF. In addition, we did not use any other, known inhibitors of the NLRP3 inflammasome, however there is none yet approved in the clinic.

In conclusion, our data demonstrate that propranolol inhibits activation of caspase‐1, indicating that its effect on IL‐1β production extends beyond transcriptional priming to the level of inflammasome activation. Although β‐adrenergic signaling has primarily been linked to modulation of NF‐κB–dependent priming, our findings suggest additional interference with upstream signaling pathways that converge on inflammasome activation. In contrast, the pyrin inflammasome, which is primarily regulated via RhoA‐dependent cytoskeletal signaling, is less likely to be directly affected by β‐adrenergic blockade.

Together, these findings position propranolol as a modulator of inflammasome‐dependent IL‐1β processing, with a mechanism most consistent with interference in TRAF6‐associated signaling and downstream NLRP3 activation thus preventing the production of IL‐1β, TNFα, and IL‐6 in monocytes of patients with FMF. Because propranolol is approved and safe for use in children, our data support the idea of conducting a clinical trial of propranolol in FMF, especially in countries where medication that specifically blocks IL‐1β is not available.

## AUTHOR CONTRIBUTIONS

All authors contributed to at least one of the following manuscript preparation roles: conceptualization AND/OR methodology, software, investigation, formal analysis, data curation, visualization, and validation AND drafting or reviewing/editing the final draft. As corresponding author, Dr Tenbrock confirms that all authors have provided the final approval of the version to be published and takes responsibility for the affirmations regarding article submission (eg, not under consideration by another journal), the integrity of the data presented, and the statements regarding compliance with institutional review board/Declaration of Helsinki requirements.

## ROLE OF THE STUDY SPONSOR

Novaritis and the Swedish Orphan Biovitrum had no role in the study design or in the collection, analysis, or interpretation of the data, the writing of the article, or the decision to submit the article for publication. Publication of this article was not contingent upon approval by Novaritis or the Swedish Orphan Biovitrum.

## Supporting information


**Disclosure Form**:

**Table S1**. Study participants
**Fig. S1**: IL‐1b release (ELISA) upon LPS+ATP stimulation in presence of increasing concentrations of Propranolol. Enclosed data are acquired from two independent healthy donors (each data point represents one donor).
**Figure S2. Propranolol treatment suppresses IL‐1β and caspase‐1 in human PBMCs from healthy donors**. Human PBMCs were stimulated for 4 hours with LPS (50 ng/mL) or LPS and ATP (2 mol/mL, added for the last 0.5 hours of stimulation). Where indicated cells were additionally treated with propranolol (100 μM) 0.5 hours prior to LPS stimulation. Subsequently, PBMCs were lysed and analyzed by Western blot using antibodies against caspase‐1, IL‐1β, and β‐actin.
**Figure S3**. **CREB‐deficient macrophages show reduced levels of IL‐1β**. Peritoneal macrophages from wild type and CREB‐deficient mice were primed with 50 ng/mL LPS for 8 hours and then treated with 600 μg/ml alum for an additional 8 hours. Supernatants were analyzed using IL‐1β ELISA. The data is presented as means, and the *p*‐value calculated using a parametric two‐sided *t*‐test.
**Figure S4**. **Additional measurements in *ApoE*** ^***−/−***^ **mice treated with propranolol**. (**A**) *ApoE* ^*−/−*^ mice received a high‐fat diet for 12 weeks, with or without additional propranolol treatment (Prop.) via the drinking water where indicated. (**B**‐**E**) Body weight, plaque frequencies, and size in the thoracic (**C**) and abdominal aorta (**D**) as well as in the aortic roots (**E**), were compared. The means ± SD for males (m), females (f) and combined data are shown. Analysis was done using two‐way ANOVA with Sidak's multiple comparison test.
