# Supplementary material for: Exploratory Analysis of the Inhibitory Effects of Propranolol on NLRP3 and Pyrin Inflammasomes
Source: ACR Open Rheumatol. 2026 Aug 23;8(8):e90133. doi: 10.1002/acr2.90133 (PMC13500453; doi:10.1002/acr2.90133)
Supplement: Supplementary file 2 [file ACR2-8-e90133-s001.docx]

**Supplemental tables and figures**

**Supplemental tables**

**Table S1.** Study participants

|  | **FMF patients** (n=6) | **Healthy controls** (n=6) |
| --- | --- | --- |
| **Demographics** |  |  |
| Age in years; median (range) | 16 (8-26) | 32 (21-43) |
| Sex, f/m | 2/4 | 3/3 |
| **Clinical parameters** |  |  |
| MEFV mutation | M694V^hom^ (6/6) | n.a. |
| Disease activity | inactive (6/6) | n.a. |
| **Medication** |  | none |
| Colchicine | 6/6 | --- |
| Canakinumab | 2/6 | --- |

FMF: familial Mediterranean fever; hom: homozygous

**Supplemental figures**

**Human monocytes**

**Fig.S1:** IL-1b release (ELISA) upon LPS+ATP stimulation in presence of increasing concentrations of Propranolol. Enclosed data are acquired from two independent healthy donors (each data point represents one donor).

Depicted below are the Western Blots of all experiments:

**Donor 1**

**Donor 2**

**Donor 3**

**Figure S2. Propranolol treatment suppresses IL-1β and caspase-1 in human PBMCs from healthy donors.** Human PBMCs were stimulated for 4 hours with LPS (50 ng/mL) or LPS and ATP (2 mol/mL, added for the last 0.5 hours of stimulation). Where indicated cells were additionally treated with propranolol (100 µM) 0.5 hours prior to LPS stimulation. Subsequently, PBMCs were lysed and analyzed by Western blot using antibodies against caspase-1, IL-1β, and β-actin.

**Figure S3**. **CREB-deficient macrophages show reduced levels of IL-1β.** Peritoneal macrophages from wild type and CREB-deficient mice were primed with 50 ng/mL LPS for 8 hours and then treated with 600 µg/ml alum for an additional 8 hours. Supernatants were analyzed using IL-1β ELISA. The data is presented as means, and the *p*-value calculated using a parametric two-sided *t*-test.

**Figure S4**. **Additional measurements in *ApoE^-/-^* mice treated with propranolol.** (**A**) *ApoE^-/-^* mice received a high-fat diet for 12 weeks, with or without additional propranolol treatment (Prop.) via the drinking water where indicated. (**B**-**E**) Body weight, plaque frequencies, and size in the thoracic (**C**) and abdominal aorta (**D**) as well as in the aortic roots (**E**), were compared. The means ± SD for males (m), females (f) and combined data are shown. Analysis was done using two-way ANOVA with Sidak’s multiple comparison test.
